# Sellar Mass in 2 Patients With Acute-Onset Headache and Visual Symptoms: Not Your Usual Pituitary Adenoma

**DOI:** 10.1016/j.aace.2023.09.004

**Published:** 2023-09-29

**Authors:** Run Yu

**Affiliations:** Division of Endocrinology, UCLA David Geffen School of Medicine, Los Angeles, California

**Keywords:** sellar mass, acute-onset headache and visual symptoms, atypical teratoid/rhabdoid tumor, sellar compression symptoms

## Abstract

**Background/Objective:**

Clinical diagnosis of rare aggressive sellar malignancies requires a high index of suspicion. The objective was to report 2 patients with primary sellar atypical teratoid (AT)/rhabdoid tumor (RT) who presented with acute-onset headache and visual symptoms.

**Case Report:**

Patient 1 was a 45-year-old woman who presented with 3 weeks of headache and 1 week of eye pain and diplopia. Magnetic resonance imaging (MRI) identified a 2.2-cm sellar mass. Pituitary hormone testing showed elevated prolactin and suppressed luteinizing hormone, follicle-stimulating hormone, and estradiol levels. Patient 2 was a 32-year-old woman who presented with 1 month of headache and 1 week of diplopia. MRI showed a 2.1-cm sellar mass. Hormonal test results were reportedly unremarkable. Both patients did not have a significant medical history. They each underwent transsphenoidal resection. Surgical histology and molecular studies were consistent with primary sellar AT/RT. After surgery, patient 1 developed bilateral blindness and was lost to follow-up. Patient 2 developed hypopituitarism; her visual symptoms improved temporarily but recurred 2 weeks later. Pituitary MRI showed sellar recurrence. She underwent further debulking, but the tumor recurred promptly again. Despite radiation therapy, she died 4 months after the original presentation.

**Discussion:**

AT/RT appears to be the most aggressive sellar malignancy.

**Conclusion:**

Based on the 2 cases presented and the literature, I conclude that rapidly progressive headache with subsequent visual impairment in women with large sellar masses is almost pathognomonic of sellar AT/RT.

**Highlights**

• Atypical teratoid (AT)/rhabdoid tumor (RT) is the most aggressive sellar malignancy
• Rapid growth of sellar AT/RT causes rapidly progressive sellar compression symptoms
• Imaging features of sellar AT/RT are not specific
• Rapidly progressive sellar compression symptoms are suggestive of sellar AT/RT

**Clinical Relevance**

Rapidly progressive headache with subsequent rapidly progressive visual impairment in female patients with a large sellar mass that becomes alarming to patients after approximately 1 month and 1 week, respectively, is possibly pathognomonic of sellar atypical teratoid/rhabdoid tumor.

## Introduction

Although there are vascular and other apparent causes of sellar masses, most sellar masses are neoplasms.^1^^,^^2^ Rarely, aggressive malignancies can arise in or metastasize to the sella. There are no reliable imaging features to differentiate aggressive sellar malignancies from pituitary adenoma.^3^^,^^4^ Acute-onset headache and visual changes have been suggested as a clue of aggressive sellar malignancies, such as primary sellar atypical teratoid(AT)/rhabdoid tumor (RT).^5^^,^^6^ In this report, 2 patients with sellar AT/RT are described to provide further support to the notion that acute-onset headache and visual changes are an important and reliable clue to lead to the diagnosis of aggressive sellar malignancies. The author was the attending endocrinologist of both cases when they were hospitalized in our tertiary center.

## Case Report

**Figure 1:**
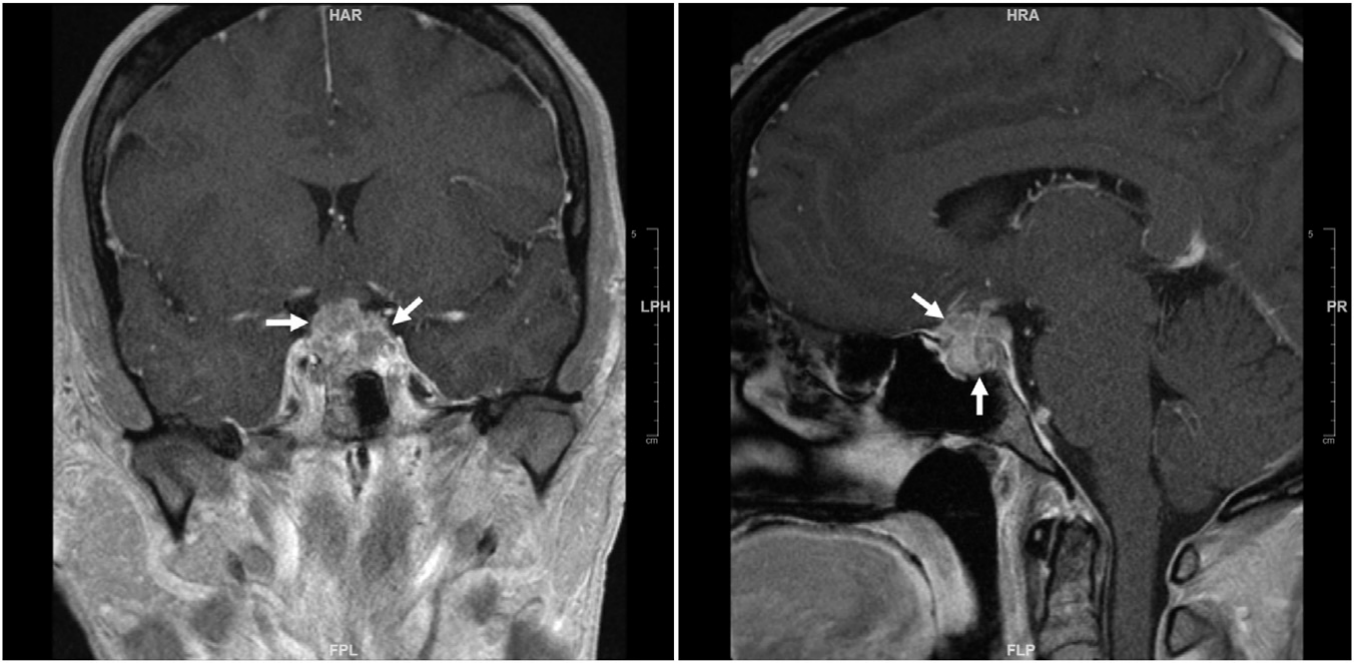
Pituitary magnetic resonance imaging of patient 1 at initial presentation. Left, coronal view; right, sagittal view. The tumor invaded into the right cavernous sinus, circumferentially encased the cavernous right internal carotid artery, abutted the left cavernous sinus and the left internal carotid artery, and slightly displaced the optic chiasm. Arrows indicate the tumor.

Patient 1 was a 45-year-old woman who presented with 3 weeks of headache and 1 week of vision symptoms. Severe right-sided headache and eye pain had started abruptly. She went to an outside hospital, where she was diagnosed with cranial nerve palsy and prescribed with corticosteroids and antivirals. Her symptoms did not improve with the treatment, and she developed binocular oblique diplopia. She then saw a neurologist by telehealth, who advised her to go to the emergency room of our tertiary center. The patient had no significant medical history. Physical examination showed binocular diplopia, which improved with left head tilt. Brain magnetic resonance imaging (MRI) identified an enhancing 16 mm × 15 mm partly sellar and partly suprasellar mass. Pituitary MRI on the same day of the brain MRI confirmed the mass, which measured 19 mm × 22 mm × 18 mm, extending from the sella to the suprasellar cistern and invading the right cavernous sinus (Fig. 1). She was thought to have a pituitary macroadenoma and was referred to endocrinology. Endocrine review of systems was only positive for hot flashes. Hormone testing showed normal levels of cortisol, thyroxine, and IGF-I but moderately elevated prolactin and suppressed luteinizing hormone, follicle-stimulating hormone, and estradiol levels (Table 1). The patient was treated with dexamethasone by neurosurgery and underwent transsphenoidal sella exploration. Only partial resection was achieved. Histologic examination of surgical specimens showed atypical epithelioid cells arranged in sheets and cords in a myxoid background. Frequent mitotic figures were identified with a Ki-67 labeling index of 40% to 70%. Immunostain revealed SMARCB1 deficiency (loss of INI1 expression). Methylation-based tumor profiling results were consistent with AT/RT, MYC subgroup, World Health Organization (WHO) grade 4.

**Table 1:** Patient 1’s Morning Pituitary-Related Hormone Levels at Presentation

Hormone	Reference range	Value
TSH	0.3 to 4.7 mcIU/mL	1.4
Free T4	0.8 to 1.7 ng/dL	1.1
LH	1 to 15 mIU/mL	0.3
FSH	1 to 8 mIU/mL	1.4
Estradiol	20 to 220 pg/mL	29
Prolactin	3 to 23.1 ng/mL	51.8
ACTH	4 to 48 pg/mL	29
Cortisol	8 to 25 mcg/dL	9
IGF-I	52 to 328 ng/mL	164
IGF-I Z-score	−2.0 to 2.0 SD	0.3

Abbreviations: ACTH = adrenocorticotropic hormone; FSH = follicle-stimulating hormone; LH = luteinizing hormone; T4 = thyroxine; TSH = thyroid-stimulating hormone.

After surgery, the patient developed transient diabetes insipidus and persistent bilateral blindness, which did not respond to high-dose corticosteroid. Chemotherapy and radiation therapy were planned but postponed by the patient as she was overwhelmed by the rapid deterioration of her health. She was then lost to follow-up.

Patient 2 was a 32-year-old woman with a recent diagnosis of AT/RT who transferred her care to our tertiary center. Four weeks before the transfer, she had presented with 1 month of severe headache and 1 week of diplopia to an outside hospital. MRI reportedly showed a 2.1-cm enhancing partly sellar and partly suprasellar mass. She was initially diagnosed with pituitary macroadenoma. Hormonal test results were reportedly unremarkable. She underwent transsphenoidal tumor resection at the outside hospital. Near total resection of the mass was achieved. Histologic examination of surgical specimens showed a densely cellular neoplasm composed of cells with round nuclei and prominent nucleoli and very high nuclear-to-cytoplasmic ratios. Mitotic activity was high with a Ki-67 labeling index of >80%. Immunostain revealed SMARCB1 deficiency (loss of INI1 expression). Methylation-based tumor profiling results were consistent with AT/RT, MYC subgroup, WHO grade 4. After surgery, her headache and visual symptoms improved temporarily, and she reportedly developed adrenal insufficiency and hypothyroidism and began treatment with corticosteroid and levothyroxine. Two weeks after surgery, headache recurred and she developed progressive bilateral vision loss. She was asked by the outside hospital to transfer her care to our tertiary center.

**Figure 2:**
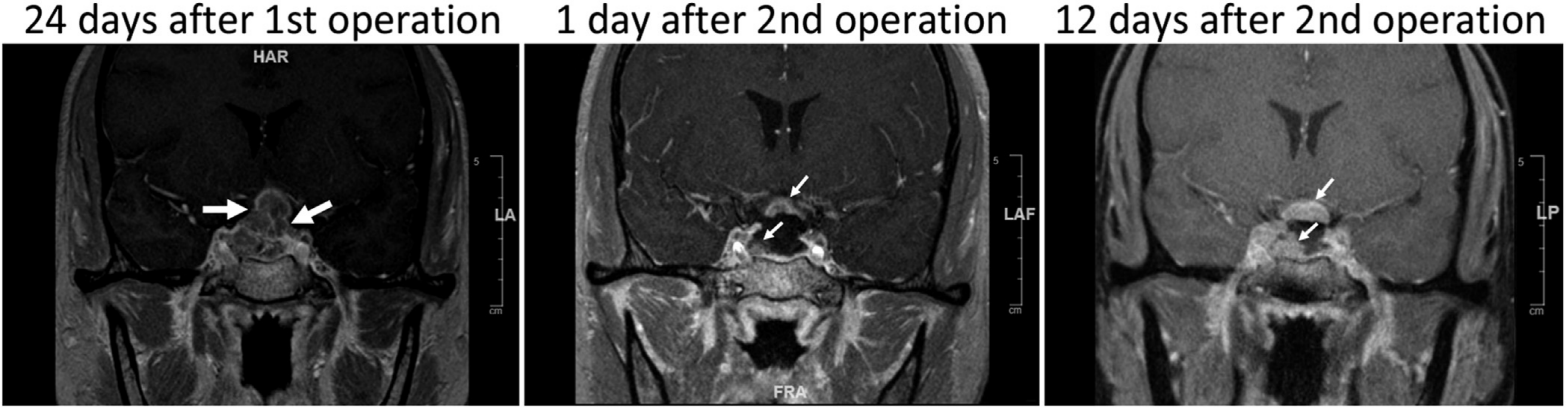
Pituitary magnetic resonance imaging of patient 2 after the first transsphenoidal operation. Left, 24 days after the first transsphenoidal operation. The tumor splayed and compressed the optic chiasm with associated edema of the optic tracts, optic chiasm, and prechiasmatic optic nerves. There was mass effect on the right cavernous sinus and dural thickening along the planum sphenoidale. Middle and right, 1 and 12 days after the second operation, respectively. During the 11 days, the residual tumor in the right sella grew from 8 mm × 6 mm to 18 mm × 11 mm, and the residual tumor in the suprasellar cistern involving the optic chiasm grew from 3 to 6 mm in maximum thickness. Arrows indicate the tumor.

At our tertiary center, she was referred to endocrinology. She had no significant medical history. Physical examination showed right-sided peripheral vision loss and left-sided blurry vision. Pituitary MRI showed postsurgical changes and a lobulated partly sellar and partly suprasellar enhancing mass measuring 24 mm × 14 mm × 16 mm (Fig. 2). Hormonal testing showed hypopituitarism (Table 2). She was treated with stress-dose corticosteroid and the levothyroxine dose was increased from 50 to 100 mcg daily. She underwent further debulking, which removed most of the recurrent tumor (Fig. 2). Histology of surgical specimens confirmed AT/RT. Her headache and vision briefly improved, but she developed persistent diabetes insipidus and started desmopressin. Twelve days after the second operation, while awaiting radiation and chemotherapy, she suffered sudden complete left vision loss. MRI showed that, in merely 11 days, the residual tumor had grown significantly (Fig. 2). In addition, there was nodular enhancement within the left internal auditory canal, concerning for leptomeningeal spread or metastasis. The patient received craniospinal radiation with sella boost. Although the residual sellar mass shrank, the patient developed extensive cervical, thoracic, and lumbar spine leptomeningeal metastasis with distinct nodules. Although chemotherapy was planned, she developed massive pulmonary embolism and shock and died 4 months after original presentation.

**Table 2:** Patient 2’s Pituitary-Related Hormone Levels

Hormone	Reference range	Morning value 3 wk after the first operation	Evening value 12 d after the second operation
TSH	0.3 to 4.7 mcIU/mL	0.09	<0.02
Free T4	0.8 to 1.7 ng/dL	0.50	1.20
Total T3	85 to 185 ng/dL	40	…
LH	1 to 15 mIU/mL	<0.3	<0.3
FSH	1 to 8 mIU/mL	<0.3	<0.3
Prolactin	3 to 23.1 ng/mL	1.4	<0.5
ACTH	4 to 48 pg/mL	<2	<2
Cortisol	8 to 25 mcg/dL	0.5	16^a^
IGF-I	52 to 328 ng/mL	…	32
IGF-I Z-score	−2.0 to 2.0 SD	…	−2.9

Abbreviations: ACTH = adrenocorticotropic hormone; FSH = follicle-stimulating hormone; LH = luteinizing hormone; T3 = triiodothyronine; T4 = thyroxine; TSH = thyroid-stimulating hormone.

a The patient was taking hydrocortisone.

## Discussion

Two patients with primary sellar AT/RT are reported who presented with acute-onset headache and visual symptoms. Sellar compression symptoms are caused by the expansion of sellar content (mostly due to neoplasms, such as pituitary adenoma and primary and metastatic sellar malignancies), which impinges on the surrounding structures, such as the cavernous sinus and optic chiasm.^1^^,^^2^ The sella and its surrounding structures are plastic organs; how they react to sellar content expansion depends largely on the chronicity of the expansion. When the expansion is slow or subacute, the sella remodels itself to enlarge its dimensions and the optic chiasm often scallops upwards; these adaptive changes are reversible upon removal of the culprit sellar lesion.^7^^,^^8^ Headache caused by pituitary adenomas usually lasts longer than a year, and visual changes last for many months before patients present to their physicians for these symptoms.^9^^,^^10^ When the sellar content expansion is acute, such as in sellar AT/RT, the sella and its surrounding structures only have little time to adapt; therefore, the sellar compression symptoms manifest in acute and progressive headache and visual impairments.^5^^,^^6^ The time courses of headache and visual impairments of the 2 cases described here are typical for AT/RT: approximately 1 month and 1 week, respectively, before the symptoms are severe enough that patients need to seek medical attention.^5^^,^^6^

AT/RT is a very aggressive and poorly differentiated central nervous system malignancy. Most AT/RT cases occur in children, but AT/RT can affect adults, and the most common affected site in adults is the sella.^6^^,^^11^ Most adult patients with sellar AT/RT are woman, and their prognosis is relentlessly poor despite surgical resection, radiation, or chemotherapy. There has been significant progress in the molecular diagnosis of AT/RT in the last decade. Loss of either INI1 protein (*SMARCB1* gene) or BRG1 protein (*SMARCA4* gene) can be used as a molecular marker of AT/RT, but these 2 markers are not uniquely specific for AT/RT.^12^ Recently, tumor DNA methylation profiling has been used as a sensitive and specific molecular method to diagnose AT/RT and to further classify it.^13^^,^^14^ The tumors in the 2 patients in this report exhibit loss of INI1 protein and are classified by DNA methylation into the MYC subgroup, WHO grade 4, typical for adult sellar AT/AT. Preoperative diagnosis of AT/RT, however, has been lagging behind; as shown in the 2 cases here, most cases of sellar AT/RT are tentatively diagnosed with pituitary macroadenoma due to the lack of specific hormonal or imaging features of AT/RT.^5^^,^^6^ The typical clinical features of sellar AT/RT, however, have made this author postulate that AT/RT can now be reliably diagnosed before surgery. The salient clinical features of AT/RT are rapidly progressive headache with subsequently developed and also rapidly progressive visual impairment in female patients with a large sellar mass that become alarming to patients after approximately 1 month and 1 week, respectively.^5^^,^^6^ Other sellar malignancies usually are not as aggressive as AT/RT. Primary sellar lymphomas exhibit indolent sellar compression symptoms, similar to those caused by pituitary adenomas.^15^ Most sellar metastatic malignancies present with pituitary functional deficiencies, such as diabetes insipidus and panhypopituitarism, not with sellar compression symptoms.^16^ Even small cell lung cancer metastasized to the sella presents with less acute sellar compression symptoms and more prominent pituitary functional deficiencies.^17^ Early recognition of AT/RT is important because futile surgical interventions could be avoided and patients and family members could be better prepared for the relentless prognosis.

## Conclusion

These 2 cases demonstrate that acute-onset headache and visual changes are highly suggestive of an aggressive sellar lesion. Specifically, rapidly progressive headache with subsequent visual impairment in female patients with a large sellar mass that becomes intolerable to patients after approximately 1 month and 1 week, respectively, is possibly pathognomonic of sellar AT/RT.

## Disclosure

The author has no multiplicity of interest to disclose.

## References

[1] Famini P., Maya M.M., Melmed S. (2011). Pituitary magnetic resonance imaging for sellar and parasellar masses: ten-year experience in 2598 patients. J Clin Endocrinol Metab.

[2] Schwetye K.E., Dahiya S.M. (2020). Sellar tumors. Surg Pathol Clin.

[3] Jipa A., Jain V. (2021). Imaging of the sellar and parasellar regions. Clin Imaging.

[4] Ugga L., Franca R.A., Scaravilli A. (2023). Neoplasms and tumor-like lesions of the sellar region: imaging findings with correlation to pathology and 2021 WHO classification. Neuroradiology.

[5] Lev I., Fan X., Yu R. (2015). Sellar atypical teratoid/rhabdoid tumor: any preoperative diagnostic clues?. AACE Clin Case Rep.

[6] Major K., Daggubati L.C., Mau C., Zacharia B., Glantz M., Pu C. (2022). Sellar atypical teratoid/rhabdoid tumors (AT/RT): a systematic review and case illustration. Cureus.

[7] Raghu A.L.B., Flower H.D., Statham P.F.X., Brennan P.M., Hughes M.A. (2020). Sellar remodeling after surgery for nonfunctioning pituitary adenoma: intercarotid distance as a predictor of recurrence. J Neurol Surg B Skull Base.

[8] Danesh-Meyer H.V., Yoon J.J., Lawlor M., Savino P.J. (2019). Visual loss and recovery in chiasmal compression. Prog Retin Eye Res.

[9] Gondim J.A., de Almeida J.P., de Albuquerque L.A., Schops M., Gomes E., Ferraz T. (2009). Headache associated with pituitary tumors. J Headache Pain.

[10] Jahangiri A., Lamborn K.R., Blevins L., Kunwar S., Aghi M.K. (2012). Factors associated with delay to pituitary adenoma diagnosis in patients with visual loss. J Neurosurg.

[11] Chan V., Marro A., Findlay J.M., Schmitt L.M., Das S. (2018). A systematic review of atypical teratoid rhabdoid tumor in adults. Front Oncol.

[12] Louis D.N., Perry A., Reifenberger G. (2016). The 2016 World Health Organization classification of tumors of the central nervous system: a summary. Acta Neuropathol.

[13] Johann P.D., Erkek S., Zapatka M. (2016). Atypical teratoid/rhabdoid tumors are comprised of three epigenetic subgroups with distinct enhancer landscapes. Cancer Cell.

[14] Koelsche C., von Deimling A. (2022). Methylation classifiers: brain tumors, sarcomas, and what’s next. Genes Chromosomes Cancer.

[15] Caputo M., Prencipe N., Bisceglia A. (2020). Primary pituitary lymphoma as rare cause of a pituitary mass and hypopituitarism in adulthood. Endocr Pract.

[16] Javanbakht A., D’Apuzzo M., Badie B., Salehian B. (2018). Pituitary metastasis: a rare condition. Endocr Connect.

[17] Liu X., Wang R., Li M., Chen G. (2021). Pituitary metastasis of lung neuroendocrine carcinoma mimicking pituitary adenoma: case report and literature review. Front Endocrinol (Lausanne).

